# Comparison of dry and wet deposition of particulate matter in near-surface waters during summer

**DOI:** 10.1371/journal.pone.0199241

**Published:** 2018-06-21

**Authors:** Yanan Wu, Jiakai Liu, Jiexiu Zhai, Ling Cong, Yu Wang, Wenmei Ma, Zhenming Zhang, Chunyi Li

**Affiliations:** 1 College of Nature Conservation, Beijing Forestry University, Beijing, China; 2 Institute of Wetland Research, Chinese Academy of Forestry, Beijing, China; Zhongnan University of Economics and Law, CHINA

## Abstract

Atmospheric particulate matter (PM) deposition which involves both dry and wet processes is an important means of controlling air pollution. To investigate the characteristics of dry and wet deposition in wetlands, PM concentrations and meteorological conditions were monitored during summer at heights of 1.5 m, 6 m and 10 m above ground level at Cuihu Wetland (Beijing, China) in order to assess the efficiency of PM2.5 (particles with an aerodynamic size of <2.5 μm) and PM10 (particles with an aerodynamic size of <10 μm) removal. The results showed: Daily concentrations of PM, dry deposition velocities and fluxes changed with the same variation trend. The daily average deposition velocity for PM10 (3.19 ± 1.18 cm·s^–1^) was almost 10 times that of PM2.5 (0.32 ± 0.33 cm·s^–1^). For PM2.5, the following dry deposition fluxes were recorded: 10 m (0.170 ± 0.463 μg·m^–2^·s^–1^) > 6 m (0.007 ± 0.003 μg·m^–2^·s^–1^) > 1.5 m (0.005 ± 0.002 μg·m^–2^·s^–1^). And the following deposition fluxes for PM10 were recorded: 10 m (2.163 ± 2.941 μg·m^–2^·s^–1^) > 1.5 m (1.565 ± 0.872 μg·m^–2^·s^–1^) > 6 m (0.987 ± 0.595 μg·m^–2^·s^–1^). In the case of wet deposition, the relative deposition fluxes for PM2.5 and PM10 were 1.5 m > 10 m > 6 m, i.e. there was very little difference between the fluxes for PM2.5 (0.688 ± 0.069 μg·m^–2^·s^–1^) and for PM10 (0.904 ± 0.103 μg·m^–2^·s^–1^). It was also noted that rainfall intensity and PM diameter influenced wet deposition efficiency. Dry deposition (63%) was more tilted towards removing PM10 than was the case for wet deposition (37%). In terms of PM2.5 removal, wet deposition (92%) was found to be more efficient.

## Introduction

The frequent occurrence of particulate matter (PM) pollution has led to many problems [1,2]. The emission of PM is one of the most important factors affecting climate [2] and health [3,4]. The presence of PM2.5 is thought to have caused premature mortality in 1.27 million individuals in China [5,6]. Likewise, PM10 may increase the risk of premature death [7] due to cardiovascular [8] and respiratory diseases [9].

Atmospheric deposition is an important means of controlling air pollution [10]. Atmospheric PM deposition involves both dry and wet processes. Dry deposition refers to the deposition of particles or gases from the atmosphere through the direct delivery of mass to the surface (i.e. via non-precipitation) [11]. On the other hand, wet processes are often referred to as rain or snow scavenging [12], with rain scavenging PM being generally classified as ‘rainout particles’ (serving as cloud-condensation nuclei or undergoing capture by cloud water) and as ‘washout’ (i.e. removal of below-cloud particles by raindrops as they fall) [13].

Studies have confirmed that dry deposition has the capacity to remove PM [14–16] and that the process of dry deposition is influenced by spatial fluctuations, surface-type differences, temporal changes, diurnal variations and meteorological conditions [14]. Wet deposition is also an important mechanism for reducing air pollution by the removal of PM [15]. A study showed that wet deposition accounted for 54–71% of PM1–20 (particles with 1–20 μm diameter) deposition and it accounted for 76–86% of PM0.5–20 (particles with 0.5–20 μm diameter) deposition [12]. According to a case study carried out in Guangzhou (China), the total annual flux of wet and dry depositions, representing the combined results for PM deposition in the urban area, was 34 g·m^–2^·yr^–1^, with 50% being attributed by wet deposition [15]. Therefore, dry and wet depositions can be regarded as important pathways for the elimination of PM from ambient air.

Wetlands are important ecological systems that perform vital ecological functions. A number of studies have demonstrated that wetlands play a role in reducing PM [17,18]. Considerable research attention has been focused on dry deposition as well as comparisons between various surface types [17,18]. Space–time variation has also been the focus of some studies. For example, PM2.5 and PM10 dry deposition fluxes at 10 m were higher than those measured at 6 m [16]. And deposition fluxes measured during the dry period (1.03 μg·m^–2^·s^–1^) were higher than those during the wet period (0.003 μg·m^–2^·s^–1^) and during normal humidity conditions (0.02 μg·m^–2^·s^–1^) [16]. Very little work has been carried out on the the ecological functions associated with dry and wet deposition in wetlands.

This study investigated the temporal and spatial variations in dry and wet deposition velocity in wetlands and compared the deposition fluxes of dry and wet deposition in order to assess the efficiency of PM2.5 and PM10 removal.

## Materials and methods

### Experimental sites

**Fig 1:**
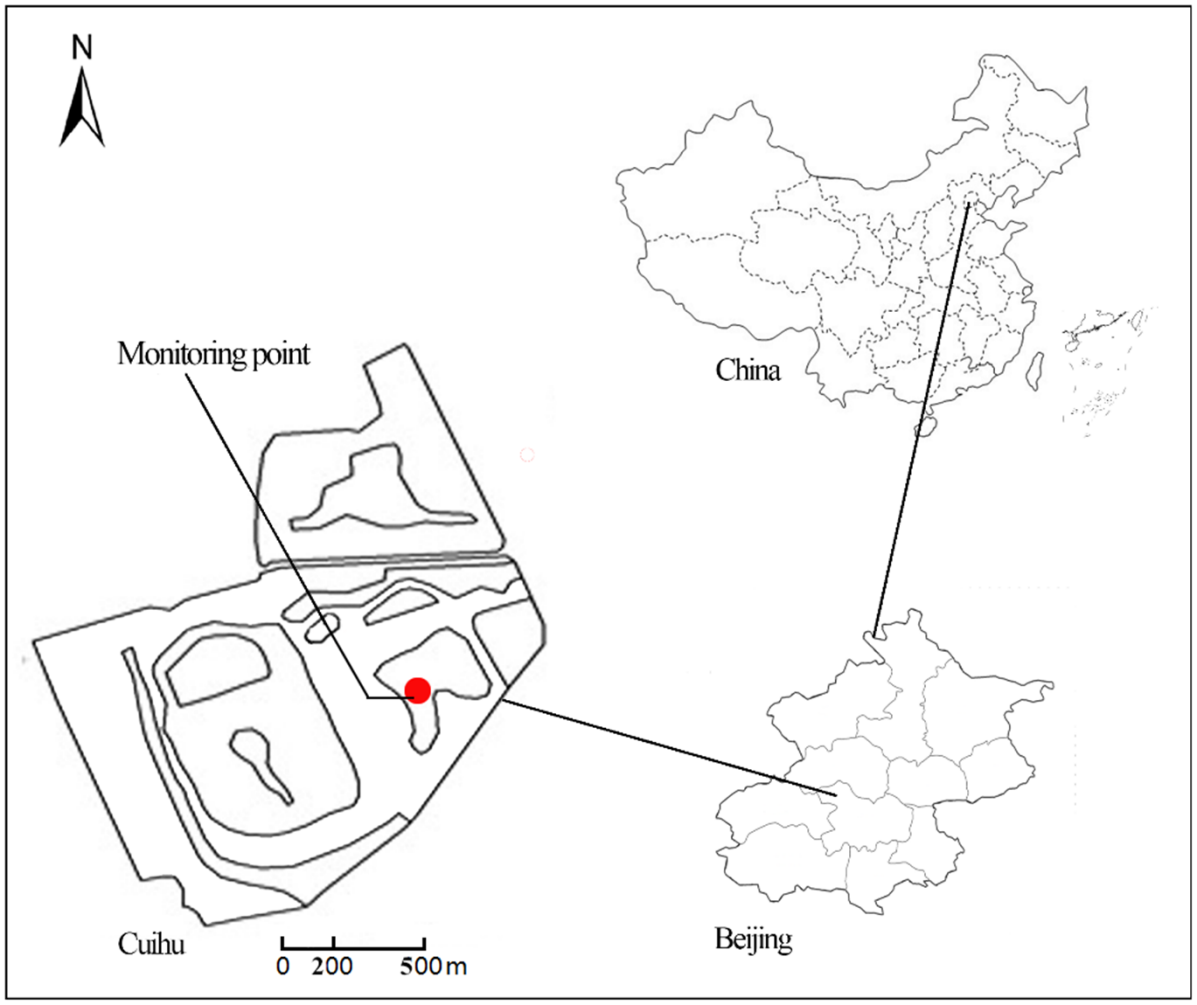
Location of study site. Reprinted from Ref. [16] under a CC BY license, with permission from Lijuan Zhu, original copyright 2016.

Cuihu National Urban Wetland Park (1.57 km^2^; 1.9 km × 1.2 km) is situated north of the Shangzhuang Reservoir, Haidian District, Beijing (Fig 1), which is in a warm temperate semi-humid monsoon climate zone. The annual average temperature is 12.38°C and the annual precipitation is 500–700 mm, 60% of which is concentrated in the wet summer period (July and August) [16]. The experiment was carried out on the “crane island” (116°19′E, 40°10′N) in Cuihu National Urban Wetland Park as shown in Fig 1.

### Experimental design

#### Ethics statement

This study has been licensed by Cuihu National Urban Wetland Park. And this study did not involve endangered or protected species.

#### Dry deposition

Particle concentration data parameters (TSP, PM10 and PM2.5) were measured using Dustmate (Turnkey Co. Ltd, UK). The using of Dustmate for collecting the PM concentration was given in previous studies [16, 17, 18] and all above these researches have successfully used this instrument. Meteorological data (wind speed, temperature and relative humidity) were collected by a weather station (Nielsen-Kellerman Co. Ltd, USA). Both the Dustmate and the weather station were mounted at three height levels, which were 1.5 m (the average plant height in the wetland), 6 m (dry deposition layer) and 10 m (stable layer) above ground level. The sampling time was from 7:00 am to 7:00 pm during summer (June–August) in 2016. The reason for the summer sampling was the rainfall concentrated in summer and we could contrast dry deposition and wet deposition on this basis.

#### Wet deposition

The sampling time was the summer (June–August) of 2016. Rainfall events of greater than 8 h of duration were chosen to represent wet deposition in order to studied the process of wet deposition under longer rainfall and compared it with the dry deposition process. Rain collectors which were located at 1.5 m, 6 m and 10 m collected rainwater every 0.5 h to provide information on volume and intensity. A Dustmate system was used to measure particle concentration during rainfall events.

### Data analysis

#### Dry deposition

Dry deposition flux can be calculated as follows [19,20]:

$$F\;=\;v_{d}∙\;\Delta c$$ (1)

Where F is deposition flux; Δc is concentration difference between constant flux layer and deposition layer and V_d_ is deposition velocity. This deposition velocity can be defined as follows:

$$\frac{1}{v_{d}}\;=\;\frac{1}{V_{C}}+\frac{1}{V_{D}}-\frac{V_{g}}{V_{C}∙V_{D}}$$ (2)

Where V_g_ is the gravitational settling speed (based on dry particle diameters); V_C_ is total transfer velocity in the constant flux layer and V_D_ is the total transfer velocity in the dry deposition layer, calculated as follows:

$$V_{g}\;=\;\rho_{p}∙C_{c}∙d_{p}^{2}∙g/18∙\mu_{a}$$ (3)

$$V_{C}\;=\;V_{C}^{\prime }+V_{g}$$ (4)

$$V_{D}\;=\;V_{D}^{\prime }+V_{g}$$ (5)

Where C_c_ is the Cunningham correlation factor; ρ_p_ is density of particles (equivalent to particle concentration); d_p_ is particle diameter; μ_a_ is air dynamic viscosity; ${\text{V}}_{\text{C}}^{\prime }$ is transfer velocity (without gravity) in the constant flux layer and ${\text{V}}_{\text{D}}^{\prime }$ is transfer velocity (without gravity) in the dry deposition layer. These can be calculated as follows:

$$C_{c}\;=\;1+\frac{2\lambda }{d_{p}}∙(1.257+0.4e^{-0.55∙d_{p}/\lambda })$$ (6)

$$V_{c}^{\prime }\;=\;\frac{1}{1-k}∙C_{d}∙u(z)$$ (7)

$$V_{D}^{\prime }\;=\;-\alpha ∙m+k^{-1}∙C_{d}∙u(z)∙{Sc}^{-1/2}+10^{-3}∙St$$ (8)

Where λ is mean free path of air (65 nm); α is a constant [10^3^ cm·s^–1^/(1 g·cm^–2^·s^–1^)]; C_d_ is the drag coefficient, calculated as follows: C_d_ = [(1.3 ± 0.3) × 10^−3^]; Sc is Schmidt number and St is Stokes number, calculated as follows:

$$S_{c}\;=\;\frac{V_{a}}{D_{B}}\;=\;\frac{\mu_{a}}{\rho_{p}}$$ (9)

$$S_{t}\;=\;\tau_{p}∙u(z)/d_{n}$$ (10)

Where V_a_ is the air kinematic viscosity; D_B_ is the Brown diffusion coefficient; u(z) is the average wind velocity; d_n_ is the dimension of the vegetation element for wetlands (d_n_ = 1) [20,21]; τ_p_ is the particle relaxation time, which can be presented as follows:

$$\tau_{p\;=\;\rho_{p}∙C_{c}∙d_{p}^{2}/18∙\mu_{a}}$$ (11)

#### Wet deposition

Wet deposition flux is calculated as follows [20,21]:

$$P_{{wet}_{i}}\;=\;{Cr}_{i}\times \frac{Rain}{t}$$ (12)

Where Pwet_i_ is the wet deposition flux, Cr_i_ is the concentration of particles, Rain is rainfall and t is the time of collection.

## Results

### Temporal and spatial variation in dry deposition

**Fig 2:**
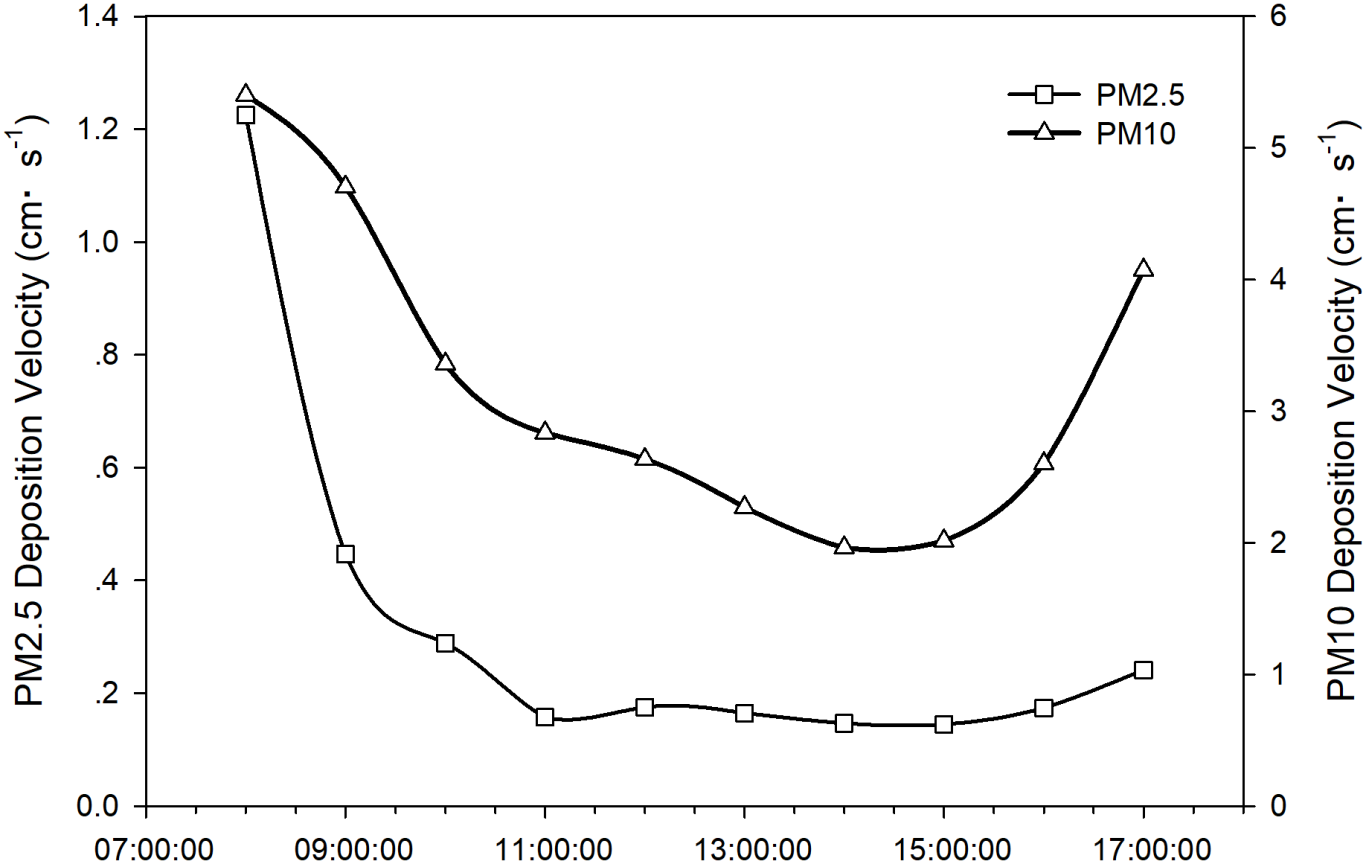
Daily variation in PM deposition velocity.

Fig 2 shows two curves relating to daily variation in PM2.5 and PM10 deposition velocity, both of which indicate similar changes. The highest value for deposition velocity was recorded at 8:00. PM2.5 deposition velocity decreased during the next three hrs and then changed in a stable manner, while PM10 deposition velocity continued to decrease until 15:00, after which there was a slight increase in PM2.5 deposition velocity and a greater increase in PM10 deposition velocity. Although the trends associated with these variations were similar, greater differences were noted between the deposition velocities of PM2.5 and PM10. The deposition velocities of PM10 were greater than those of PM2.5. The daily average deposition velocity of PM10 was 3.19 ± 1.18 cm·s^–1^, almost 10 times that of PM2.5 (0.32 ± 0.33 cm·s^–1^). Minimum PM2.5 and PM10 deposition velocities were 0.14 cm·s^–1^ and 1.96 cm·s^–1^, respectively, both of which were recorded between 14:00 and 15:00.

**Fig 3:**
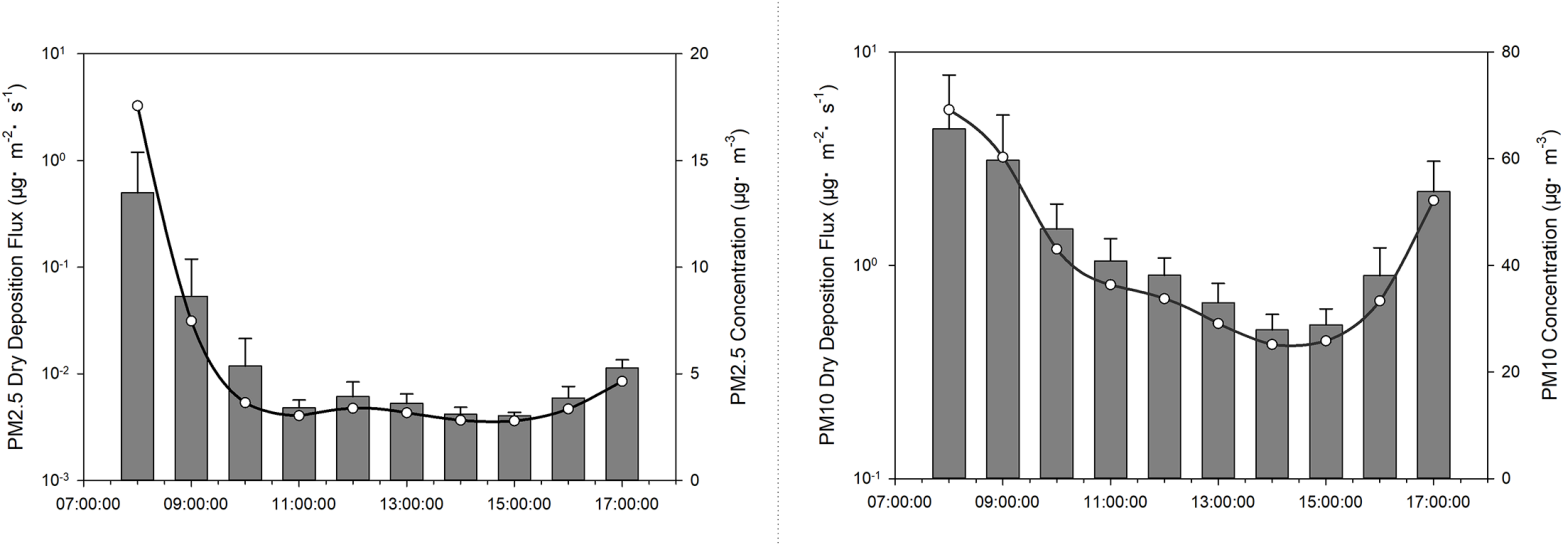
PM concentration and dry deposition flux.

The relationship between PM concentration and dry deposition flux is shown in Fig 3. These results suggest that variation trends in PM concentrations followed the same pattern as that associated with dry deposition flux. When PM concentrations were high, dry deposition flux increased; after a period of time, the concentration of PM in the air decreased, resulting in a decrease in dry deposition flux. Because of the low dry deposition flux, PM concentration again increased and accordingly the dry deposition flux increased. PM concentration and dry deposition flux thus had a mutual influence on each other. The highest PM2.5 and PM10 dry deposition flux values were recorded at 8:00, as were the concentrations of PM2.5 and PM10. Average dry deposition fluxes of PM2.5 and PM10 were 0.06 ± 0.15 μg·m^–2^·s^–1^ and 1.57 ± 1.28 μg·m^–2^·s^–1^, respectively. These results clearly indicate that the dry deposition flux of PM10 was much higher than that of PM2.5.

**Fig 4:**
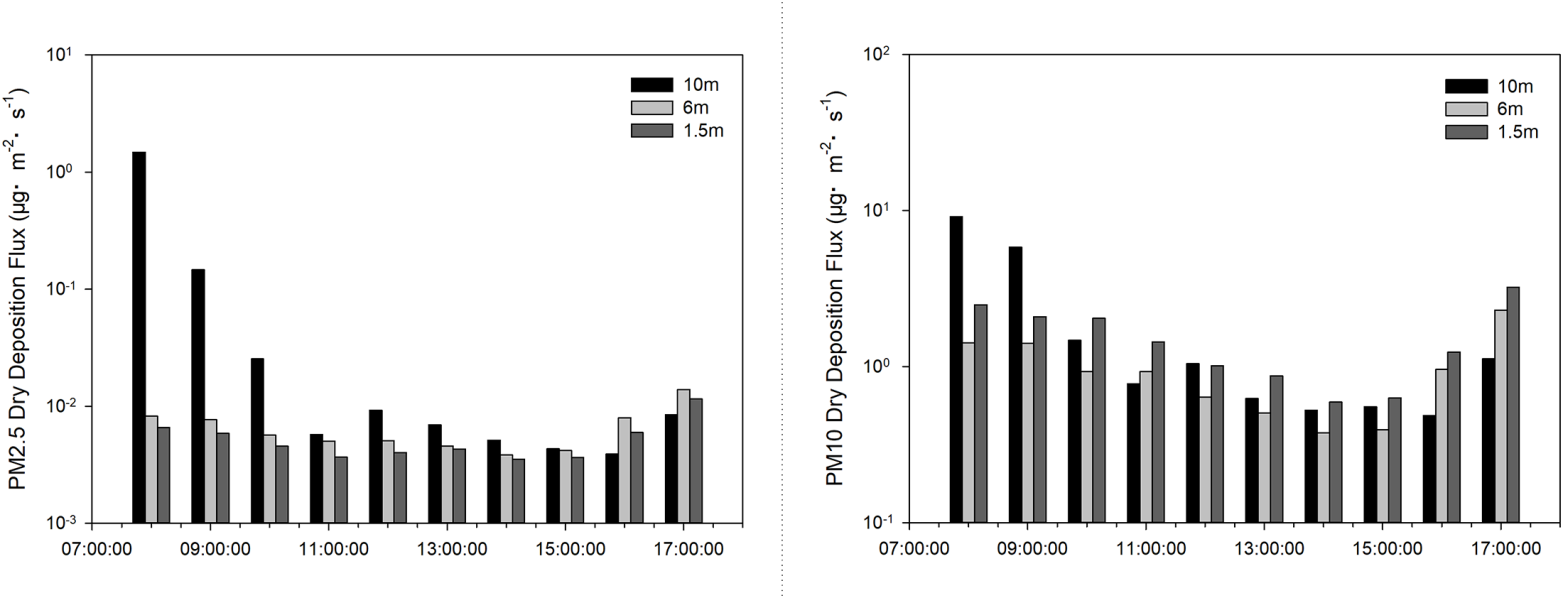
PM dry deposition flux on different height levels.

Information on the spatial variation in PM2.5 and PM10 deposition flux is summarised in Fig 4. PM2.5 deposition flux presented a downward and an upward trend, and then declined and rose again when it reached 10 m and 1.5 m, respectively. These changing tendencies resulted in an initial peak of 6 m, which later declined. The highest value occurred at 17:00 at both 6 m and 1.5 m. When it reached the height of 10 m, the highest flux occurred at 8:00. The maximum deposition flux (1.48 μg·m^–2^·s^–1^) for PM2.5 occurred at 10 m, which was considerably greater than that recorded at 6 m (0.007 ± 0.003 μg·m^–2^·s^–1^) and at 1.5 m (0.005 ± 0.002 μg·m^–2^·s^–1^). With respect to PM10, the deposition flux at 10 m initially increased and then decreased. At heights of 1.5 m and 6 m the flux pattern changed in a similar manner to that associated with PM2.5 in which the highest fluxes occurred at 1.5 m and 10 m. The highest values at the three height levels occurred at the same time, as was the case for PM2.5. However, the maximum flux was 9.18 μg·m^–2^·s^–1^, almost 6 times that recorded for PM2.5.

### Temporal and spatial variation in wet deposition

**Fig 5:**
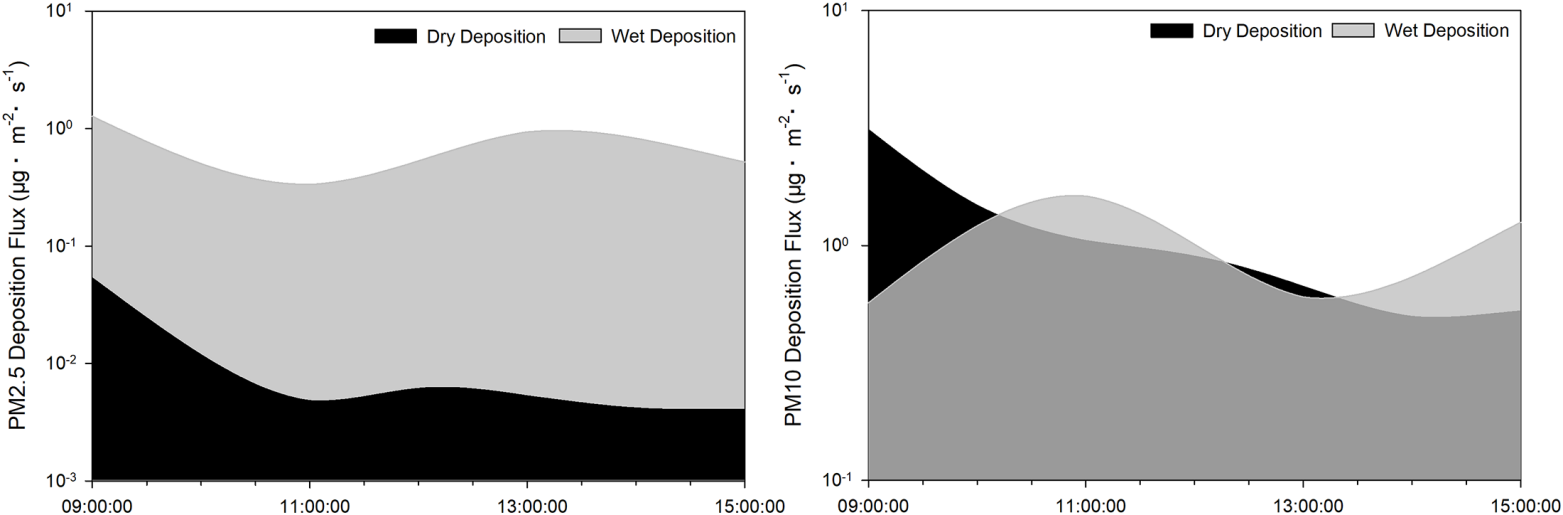
Temporal variation of dry and wet deposition.

As shown in Fig 5, the wet deposition flux curves for PM2.5 and PM10 are changeable. PM2.5 deposition flux reached a peak at the onset of rain, after which it fell sharply. The minimum flux of PM2.5 occurred after four rainy hours, when the PM10 wet deposition flux underwent an increase. Thereafter, the PM2.5 flux increased and then declined. Opposite trends were noted in the case of PM10. The PM10 deposition flux during the precipitation process thus varied in an opposite manner to that observed for PM2.5. Deposition flux of PM2.5 declined from 0.34 μg·m^–2^·s^–1^ to 1.27 μg·m^–2^·s^–1^. In the case of PM10, the maximum and minimum wet deposition fluxes were 1.62μg·m^–2^·s^–1^ and 0.57 μg·m^–2^·s^–1^, respectively. Unlike dry deposition, there was very little difference between the values for wet deposition flux for PM2.5 and PM10, being between PM2.5 (0.77 ± 0.42 μg·m^–2^·s^–1^) and PM10 (1.01 ± 0.52 μg·m^–2^·s^–1^), respectively.

**Fig 6:**
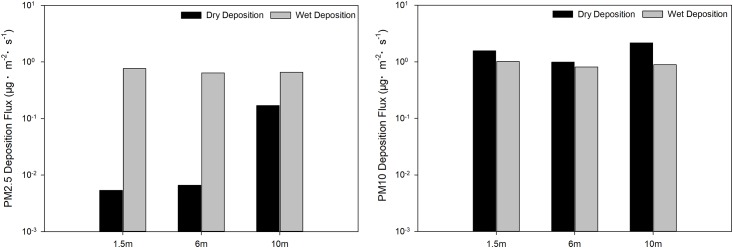
Spatial variation of dry and wet deposition.

As illustrated in Fig 6, the wet deposition flux trends (1.5 m > 10 m > 6 m) were similar for PM2.5 or PM10, with minimal differences between 1.5m, 6m and 10m. The flux of PM10 (0.904 ± 0.103 μg·m^–2^·s^–1^) was, however, slightly larger than that of PM2.5 (0.688 ± 0.069 μg·m^–2^·s^–1^).

### Comparison of dry and wet deposition

A comparison of temporal variation between dry and wet deposition is illustrated in Fig 5. PM2.5 deposition flux was changeable for wet deposition, within a range of 0.335–1.273 μg·m^–2^·s^–1^. The flux reached a maximum at the start of precipitation and fell to a minimum level after 2 h. Thereafter, the curve remained steady until 13:00, after which it declined. The changing tendency resulted in two ‘V’- shaped graphs, one positive, and the other an inverted ‘V’. In the case of dry deposition, the values for PM2.5 remained steady. The wet deposition flux (0.766 ± 0.422 μg·m^–2^·s^–1^) was nearly 60 times the dry deposition flux (0.013 ± 0.018 μg·m^–2^·s^–1^). Accordingly, wet deposition for PM2.5 was much more obvious than dry deposition. In contrast, wet deposition flux was steady and the dry deposition fluxwas changeable in the case of PM10. Dry deposition flux (1.18 ± 0.92 μg·m^–2^·s^–1^) declined during the whole process, from 3.12 to 0.50 μg·m^–2^·s^–1^ and made no big difference with wet deposition flux (1.01 ± 0.52 μg·m^–2^·s^–1^).

Variation in dry and wet deposition at different height levels is illustrated in Fig 6. In the case of PM2.5, the order of dry deposition flux is 10 m > 6 m > 1.5 m and the flux at 10 m (0.17 μg·m^–2^·s^–1^) was far greater than that measured at 6 m and 1.5 m. In the case of wet deposition, the sequence trend was as follows: 1.5 m > 10 m > 6 m. However, there were no significant differences between these values. Wet deposition flux was, however, greater than dry deposition flux at each height level. In the case of PM10, the values for dry deposition flux were 1.57 μg·m^–2^·s^–1^, 0.99 μg·m^–2^·s^–1^ and 2.16 μg·m^–2^·s^–1^ at 1.5 m, 6 m and 10 m, respectively. The flux at 10 m was highest, which was consistent with the results obtained for PM2.5. The values of PM10 wet deposition flux were 1.01 μg·m^–2^·s^–1^, 0.81 μg·m^–2^·s^–1^ and 0.89 μg·m^–2^·s^–1^ at 1.5 m, 6 m and 10 m, respectively.

## Discussion

### Meteorological factors and dry deposition

**Fig 7:**
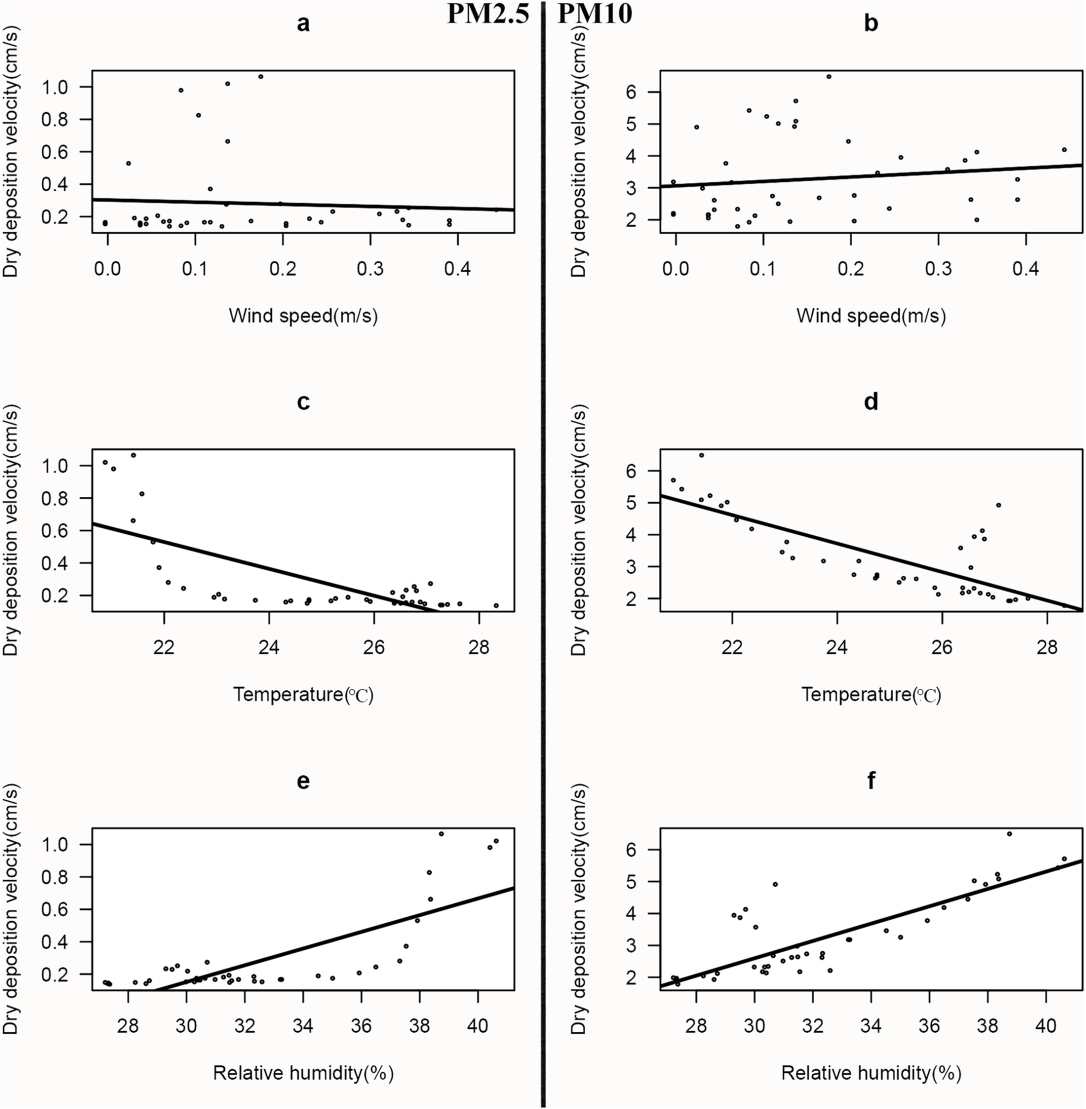
Relations between PMs dry deposition velocity and meteorological factors.

Meteorological factors, such as wind speed, temperature and relative humidity, can influence friction velocity and atmospheric stability [22]. Deposition velocity and PM concentration were also strongly affected by friction velocity and atmospheric stability [23–25]. Therefore, we can conclude that meteorological factors have an important impact on deposition velocity. The relationship between dry deposition velocity and meteorological factors was investigated by means of correlation analysis between dry deposition velocity and meteorological parameters, as illustrated in Fig 7 (at a confidence level of 95%).

The mean wind speed, temperature and relative humidity at the sampling site were 0.16 m/s, 24.99°C and 32.52%, respectively. These results indicated that there was no significant association between dry deposition velocity and wind speed of PM (Fig 7a and 7b). However, this result did not conform with the results of other studies [26,27] and the relevant explanations made by some physical models, such as firm theory and the resistance model [28–30]. Furthermore, wind speed could also influence the particles accumulation on leaves [31]. Wind speed was the strongest meteorological factor, which influenced dry deposition velocity [26] and had a positive correlation with the dry deposition velocity of TSP [22,26–29]. The results of our study may have been affected by the low wind speed conditions that prevailed during the sampling period.

The dependence of temperature on dry deposition velocity is illustrated in Fig 7c and 7d, which indicates the following correlation coefficients between dry deposition velocity and temperature: −0.72 for PM2.5 and −0.78 for PM10. Other results indicate that PM2.5 (R = 0.78; Fig 7e) and PM10 (R = 0.82; Fig 7f) deposition velocities were significantly positively correlated with relative humidity. Hence, PM deposition velocity had a significant correlation with temperature and relative humidity. PM deposition velocity was negatively correlated with temperature and positively correlated with relative humidity. Similar results were reported in previous studies, which indicated a strong negative correlation between temperature and dry deposition velocity of TSP [22] and that an increase in relative humidity could lead to a significant increase in particle deposition rate, due to the increase in particle size [32].

### Rainfall intensity and wet deposition

**Fig 8:**
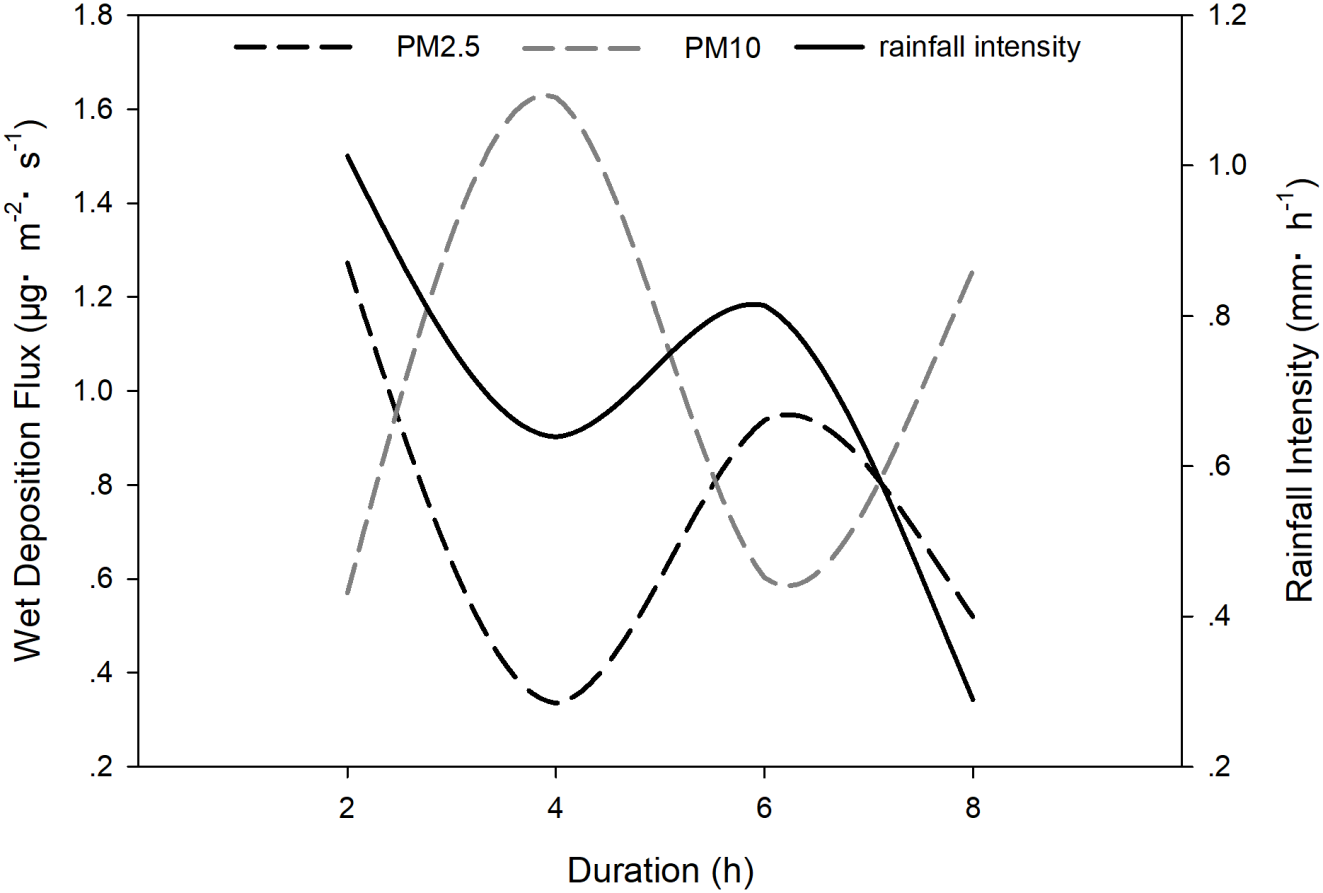
Relations between PMs wet deposition flux and rainfall intensity.

As shown in Fig 8, PM wet deposition flux and rainfall intensity were changeable during precipitation. Variation in PM2.5 deposition flux was consistent with that in rainfall intensity. In contrast, variation in PM10 deposition flux was adverse to that in rainfall intensity. Rainfall intensity thus had opposite effects on PM2.5 and PM10. There were, however, no obvious differences between wet deposition fluxes of PM2.5 and PM10.

Wet deposition can reduce air pollution by removing PM and other pollutants [33]. The efficacy of this removal is related to the duration and intensity of the precipitation event [33–36].

Results of further research have indicated a negative correlation between precipitation rates and PM2.5 concentrations [36–40]. Increases in precipitation can cause an increase in wet deposition of PM2.5 and their gaseous precursors [41]. Results obtained in our study supported the conclusion that precipitation intensity is positively correlated with PM2.5 deposition efficiency.

Our results also indicated a linear relationship between the intensity and duration of rainfall and the value of the PM10 removal coefficient [42]. The scavenging coefficient Λ(S^–1^) was considered to be the most important parameter characterising the scavenging effects of particles [43,44]. Field experiment average scavenging coefficients of PM10 for different rain intensities are listed in Table 1 [45–48]. Unlike previous studies [45–48], the changed tendency of wet deposition for PM10 had an opposite variation to rainfall intensity. This may be due to the limited range of rainfall intensity (0.29–1.01 mm/h) during the study period.

**Table 1:** Average PM10 scavenging coefficient for different rain intensity.

Rain intensity (mm/h)	Bae et al. (2001)	Baklanov (2001)	Chate (2003)	Zhao (2006)
0.2–0.5(Light Rain)	8.50E-04	1.01E-03	1.20E-03	1.60E-03
0.5–4.0(Moderate Rain)	2.18E-03	1.38E-03	1.78E-03	3.63E-03
>4.0(Heavy Rain)	5.80E-03	1.90E-03	3.40E-03	7.20E-03

### Concentration and deposition of PM

As shown in Fig 3, the concentration of PM reached a maximum at 8:00, mainly because temperature inversion limited the diffusion of pollutants during the morning [49]. Due to the high relative humidity, which caused a high density of vapour, secondary aerosols formed over wetlands in the late afternoon [50].

Variation trends in dry deposition velocities of PM2.5 were similar to those of PM10 (Fig 2), owing to the PM deposition being significantly correlated with the PM concentration [50]. Particle size had a great influence on dry deposition velocity, which tended to increase with particle size [12], i.e. the larger the particle, the higher the settling rate [51,52]. Changes in the range of velocities may be influenced by meteorological conditions, particle size distribution, particle morphology and chemical composition [12]. As shown in Figs 2 and 3, changing trends and the level of dry deposition flux followed the same laws as those of velocity since both the concentration and the deposition velocity had an impact on deposition flux [16]. As a result of the influence of gravity acceleration, deposition velocity increases with an increase in height [53]. Maximum deposition fluxes of PM2.5 and PM10 thus occurred at the height of 10 m. While the PM10 deposition fluxes of 1.5 m were higher than those of 6 m in our study. It may because of the particle dry deposition is a dynamic process, which may also be affected by spatial fluctuations, surface-type differences, temporal changes, diurnal variations and meteorological conditions [14]. And the low surface was also greatly influenced by human activity.

In the case of wet deposition, rain scavenging generally takes place via Brownian diffusion, inertial impaction, diffusiophoresis, thermophoresis and electrical charge effects [54–56]. The process of rain scavenging is affected by many factors, including raindrop size distribution and intensity, particle size distribution and concentration, the chemical and physical properties of droplets and atmospheric temperature regimes [57,58]. The PM10 wet deposition fluxes at each height level were greater than those associated with PM2.5, which were shown in Fig 6. It has been confirmed that there exists a ‘Greenfield gap’ between particles (of sizes ranging from 1 to 2 μm) during the collection process of aerosol particles [59,60]. Particles of diameters of 0.1 to 2 μm were too small to effectively get collected by inertial impaction and too large for Brownian diffusion, but the effect of inertial impaction and Brownian diffusion were much more efficient for particles of diameters of <0.1 μm and >2 μm [61]. This may explain the results that indicated greater collection efficiency for PM10 than for PM2.5. The PM2.5 wet deposition flux changed with changes in rainfall intensity during precipitation, but this was not the case for the PM10 wet deposition flux. This phenomenon was mainly caused by the sensitivity of the scavenging coefficient to the aerosol size [61] and limitation of the range of rainfall intensity.

Compared to dry deposition, wet deposition was more effective in scavenging PM2.5, a result that was supported by a previous study [12]. While a dry process can remove PM10 more effectively, the result was consistent with the conclusion that dry deposition flux was more skewed towards coarse/large particles than wet deposition flux [12].

## Conclusions

Based on this investigation, the following conclusions can be made:

Similar trends were noted for dry deposition, daily variation in concentration, deposition velocity, and flux for PM: they peaked at 8:00 and then decreased, but a slight revival was noted at 15:00. These activities therefore appeared to influence each other. It must be pointed out that the values of PM10 were higher than those of PM2.5 and the maximum occurred at 10 m. For PM2.5, the order of dry deposition fluxes was 10 m > 6 m > 1.5 m and for PM10 the flux order was 10 m > 1.5 m > 6 m. In the case of wet deposition, PM10 deposition flux values at each height level were more than PM2.5 deposition flux values, and the sequences of both PM2.5 and PM10 deposition fluxes were as follows: 1.5 m > 10 m > 6 m. In comparison with dry deposition, wet deposition was more efficient in terms of PM2.5 removal. Nevertheless, dry deposition was more tilted towards PM10 than wet deposition.

The dry deposition velocity of PM had a significant negative correlation with temperature. The correlation coefficients of dry deposition velocity and temperature were −0.72 for PM2.5 and −0.78 for PM10, and were positively correlated with relative humidity. The correlation coefficients of dry deposition velocity and relative humidity were 0.78 for PM2.5 and 0.82 for PM10. Rainfall intensity and PM diameter were important factors that influenced wet deposition efficiency. Many aspects of this process require further research.

In summary, the effects of dry and wet deposition in wetland on the concentrations of atmospheric particulates were evaluated and compared in our study. This work provided the data foundation for measures which could help improving air quality. The removal efficiency of PM2.5 by wet deposition was remarkable, indicating that it is necessary to control the air pollutant emissions during the dry weather season. What’s more, some previous studies [62–64] focus on the relationship between deposition and metals. It was surprisingly found that there was no significant correlation between metals in PM10 and rainfall except for vanadium and nickel. And It was difficult to predict metal scavenging by rainfall characteristics [62]. These processes are not considered in our study, therefore, more attention should be paid to further investigations and field studies about the metals change during the wet deposition.
